# Experimental demonstration of a photonic reservoir computing system based on Fabry Perot laser for multiple tasks processing

**DOI:** 10.1515/nanoph-2023-0708

**Published:** 2024-03-04

**Authors:** Xingxing Guo, Hanxu Zhou, Shuiying Xiang, Qian Yu, Yahui Zhang, Yanan Han, Tao Wang, Yue Hao

**Affiliations:** State Key Laboratory of Integrated Service Networks, Xidian University, Xi’an 710071, China; State Key Discipline Laboratory of Wide Bandgap Semiconductor Technology, School of Microelectronics, Xidian University, Xi’an 710071, China

**Keywords:** photonic time delay reservoir computing, multiple tasks processing, Fabry Perot laser, multiple longitudinal modes

## Abstract

Photonic reservoir computing (RC) is a simple and efficient neuromorphic computing framework for human cortical circuits, which is featured with fast training speed and low training cost. Photonic time delay RC, as a simple hardware implementation method of RC, has attracted widespread attention. In this paper, we present and experimentally demonstrate a time delay RC system based on a Fabry Perot (FP) laser for multiple tasks processing. Here, the various tasks are attempted to perform in parallel in the multiple longitudinal modes of the FP laser. It is found that the time delay RC system based on the FP laser can successfully handle different tasks across multiple longitudinal modes simultaneously. The experimental results demonstrate the potential of the time delay RC system based on the FP laser to achieve multiple tasks processing, providing various possibilities for improving the information processing ability of neural morphology RC systems, and promoting the development of RC systems.

## 1 Introduction

Reservoir computing (RC), as a brain-inspired computational paradigm, has important application potential in the field of information processing [1], [2], [3], [4], [5], [6], [7]. The proposal of RC stems from a reflection on some limitations of traditional recurrent neural networks (RNNs) [2]. Traditional RNNs require training through backpropagation algorithms, which are relatively slow and susceptible to problems such as vanishing or exploding gradients. The new idea of RC is that it does not require training of the reservoir, only the output layer, greatly simplifying the training process of neural networks.

The characteristics of the training process give RC a unique advantage for photonic network implementation. On one hand, using a fixed network structure can avoid the need for photonic chips with fully reconfigurable topologies, significantly reducing the chip area and required circuit inputs [8]. On the other hand, compared to photonic neural networks that previously required backpropagation through external calculations, using an RC training mode without backpropagation is more suitable for photonic implementation [9]. In addition, the RC maps the low dimensional space input information into high dimensional space with the reservoir to obtain a high dimensional matrix which could present more comprehensive features. Due to the rich features in high dimensional matrices, the RC could be more flexibility to tolerance the form and error integrated photonics design and manufacturing. Besides, it can achieve computing functions based on widely used photonics devices such as semiconductor lasers, semiconductor optical amplifiers, micro ring resonators, delay lines, arrayed waveguide gratings, et al. [10], [11], [12], [13], [14], [15], [16], [17], [18].

In different implementations of photonic systems, semiconductor lasers have become promising candidates due to their advantages of low power consumption, ultra-high speed, and high bandwidth. Especially the time delay RC systems based on semiconductor lasers have received great attention. In a time delay RC systems based on semiconductor lasers, replacing a large number of physical nodes in traditional reservoirs with semiconductor lasers with feedback greatly simplify the hardware implementation difficulty of the RC system [16], [17], [18], [19], [20], [21], [22], [23], [24], [25]. Recently, the time delay RC systems based on different types of semiconductor lasers have been widely proposed. For example, in 2015, Nguimdo et al. numerically demonstrated photonic time delay RC system based on single-longitudinal mode semiconductor ring laser (SRL) with optical feedback, and two independent computational tasks are realized in parallel [26]. ln 2020, Guo et al. proposed a time delay photonic RC system with a single vertical cavity surface emitting laser (VCSEL) with feedback when two polarization modes coexistence. In such system, parallel task processing is realized, including chaotic time series prediction and waveform recognition tasks [27]. In 2021, Guo et al. demonstrated numerically a time delay RC system using a semiconductor nano laser (SNL) with double phase conjugate feedbacks, and when increased two important physical factors (including the cavity-enhanced spontaneous emission factor and the enhanced spontaneous emission coupling factor), the range of good prediction performance of the SNL-based RC system was extended [28]. In 2022, Yang et al. numerically verified an RC system based on the spin VCSEL with optical feedback and injection. It was found that, benefiting from feasible tunability and multiplexing of the left and right circularly polarized modes of the spin VCSEL, the proposed RC realized both single task processing and parallel tasks processing [24]. In 2023, Huang et al. proposed employing multiple VCSELs to construct the reservoir, boosting the performance of the RC system [25]. Obviously, these methods primarily enhance the information processing capabilities of the RC system by leveraging the inherent advantages of each laser, such as rich polarization dynamics of VCSEL, modal degrees of freedom of SRL, and the complex dynamics of multiple lasers, et al. [29], [30], [31], [32], [33].

Notably, FP laser, as a well-known type of laser, has several advantages, including extremely high frequency selectivity, high stability, ease of integration, and the ability to support multiple longitudinal modes [34], [35]. These advantages make FP laser widely applicable in various fields, including optical communication, spectral analysis, laser measurements et al. Recently, the FP laser or the multimode semiconductor lasers has been attempted to construct RC systems, and numerical studies [36], [37] and experimental verification [38] have been conducted. The majority of works in the time delay RC system based on FP laser or multimode semiconductor lasers focus on utilizing longitudinal modes to expand the number of virtual nodes within the same delay length to handle same tasks with higher processing requirements [36], [37], [38]. However, overall, the works of FP based RC systems are mainly focused on the numerical research stage, especially in the use of specific longitudinal modes for multitasking different tasks in the FP lasers, which deserves further in-depth research. This is of great significance for improving the computational efficiency of RC systems.

In this paper, we present an experimental approach for the photonic time delay RC system based on an FP laser for multiple tasks processing. The various tasks are attempted to perform in parallel in the multiple longitudinal modes of the FP laser. The remainder of this paper is organized as follows. Section 2 introduces the schematic diagram and experimental scheme of the time delay RC system based on the FP laser for multiple tasks processing. In Section 3, the experimental results are introduced in detail. Especially, the multiple tasks processing performance of the time delay RC system based on the FP laser for different injection cases and two different adjacent information processing channel intervals are considered. Besides, the performance of the RC system has been examined and compared for both the multiple tasks processing and single task processing. Additionally, the effect of frequency detuning is also considered. Finally, the conclusions are given in Section 4.

## 2 Experimental scheme

**Figure 1:**
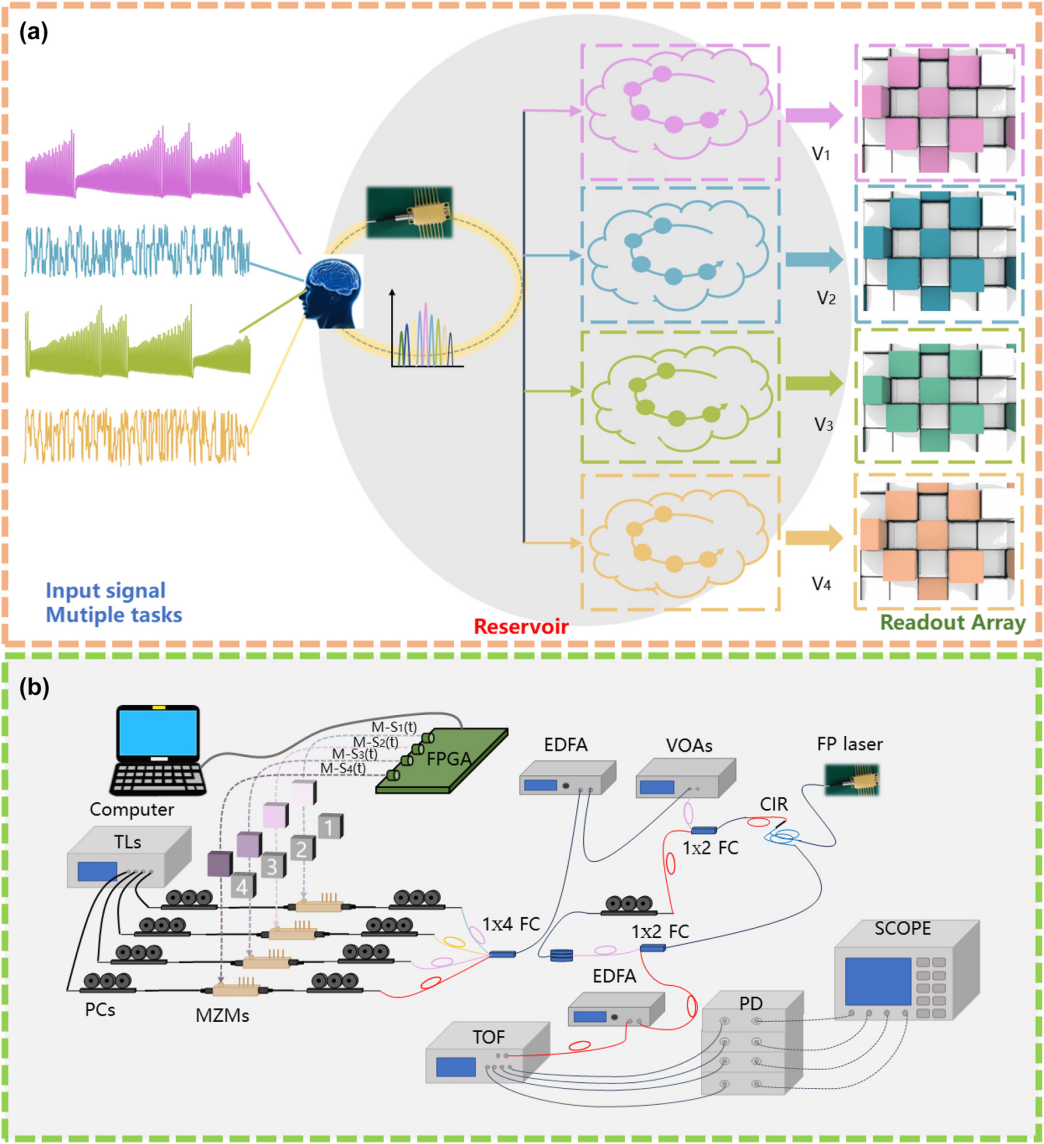
Scheme diagram. (a) The schematic diagram of the proposed time delay RC system based on the FP laser with feedback for multiple tasks processing, *V*_1_–*V*_4_: instantaneous states of four longitudinal modes in the FP laser; (b) the experimental setup of the time delay RC system based on the FP laser for multiple tasks processing. FPGA: field programmable gate array (zynq ultrascale + rfsoc zcu216); TLs: tunable semiconductor laser; 1 × 4 FC: fiber coupler with the power distribution ratio 25 %:25 %:25 %:25 %; CIR: optical circulator; VOA: variable optical attenuator; EDFA: erbium-doped optical fiber amplifier; DL: fiber delay line; PC: polarization controller; MZM: Mach–Zehnder modulator; PD: photodiode; SCOPE: oscilloscope; TOF: multi-channel tunable optical filter.

The schematic diagram of the proposed time delay RC system based on the FP laser with feedback for multiple tasks processing is displayed in Figure 1(a). In such time delay RC system, the FP laser with self-feedback light is utilized as the reservoir layer. Multiple information processing channels are realized by the multiple longitudinal modes in the FP laser. The number of virtual nodes of each channel is *n*. The interval between the adjacent virtual nodes is *θ*. The feedback delay is *τ*. The sampled period of input signal is *T*, and the formation processing rate is *R* = 1/*T*. It can be seen that, four input signals are coupled to the four longitudinal modes in the FP laser, respectively. Finally, the transient responses *E*_1*x*,2*x*,3*x*,4*x*…_ from each of the multiple channels are extracted for post-processing, respectively. In the post-processing phase, the virtual node states extracted from the multiple channels are put into multiple matrices, respectively.

The experimental setup of the time delay RC system based on the FP laser for multiple tasks processing is presented in Figure 1(b). In the experiment, a commercially available 1550 nm FP laser (BFSLD-1550-02SM-FA) is driven by a high-stability and low-noise laser diode controller. Considering the limitations of existing fibre optic platforms, we use four longitudinal modes in the FP laser for multiple processing of four signals. In addition, the FP laser used in the experiment can be described using the numerical model employed in Ref. [36]. At first, four original input signals convert to the masked input signals {M-S_1_(t), M-S_2_(t), M-S_3_(t), M-S_4_(t)} after multiplied by the binary random mask M(t) (−1, 1). The four mapped masked input signals {M-S_1_(t), M-S_2_(t), M-S_3_(t), M-S_4_(t)} are generated by a field programmable gate array (FPGA, zynq ultrascale + rfsoc zcu216), which integrates multi-channel DAC internally at the speed of 6.4 GSa/s. And then the four mapped masked input signals sent to the RF port of four electro-optical modulators (MZMs) separately. The MZMs used in the experiment to modulate the output of a multi-channel tunable semiconductor laser (TLs, TSP-400) for matching the wavelengths of the longitudinal modes of the FP laser. For each output of TLs, two polarization controllers (PCs) are sequentially used. The first PC is used to adjust to align with the modulation axis of the MZM. The second PC is adopted to control the polarization of the masked input signal. Then four input mask signals are combined into one input through a 1 × 4 fibre coupler (FC, the power distribution ratio is 25 %:25 %:25 %:25 %), and then pass through an erbium-doped optical fiber amplifier (EDFA) and a variable optical attenuator (VOA), which can adjust the power of the input mask signals. Then the input mask signals injected to the FP laser by an optical circulator (CIR). 10 % of the output of the FP laser is feedback to the FP laser by the CIR after passing through the delay line (DL) and PC. The remaining 90 % of the output of FP laser passes through a multi-channel tunable optical filter (TOF, WaveShaper 16000A). By setting the center wavelength and bandwidth of the four channels in the TOF, the output of the FP laser is divided into four channels, and then respectively into four photodetectors (PD, Ag-ilent/HP11982A) for photoelectric conversion, and finally acquired by the oscilloscope (SCOPE, Keysight DSOZ592A). Note, during the experiment, *θ* = 1/6.4 ns = 156.25 ps, *n* = 128 are fixed. Besides, for every five actual instantaneous responses, we take a virtual node every, that is, *τ* = *T* = 5 × *n* × *θ* = 100 ns. Therefore, the information processing speed of our RC system is 0.01 Gsa/s, which is slower than that in other papers [37], [39], [40], [41].

The Santa-Fe chaotic time series prediction task (denoted as S task) and the channel equalization task (denoted as N task) are used to examine the multiple tasks processing performance of the time delay RC system based on the FP laser.

On one hand, the Santa-Fe chaotic time series is recorded from a far-infrared laser operating at a chaotic state by experiment [42]. This dataset contains 9000 sample points. We take 3000 points for training and 1000 points for testing [43]. In this task, one step ahead for such a time series needs to be predicted. The completion of the Santa Fe chaotic time series prediction task requires the support of RC’s nonlinearity and memory ability. Normalized mean square error (NMSE) can be used to quantitatively evaluate the performance of this task [43]:

(1) $$\text{NMSE}=\frac{1}{L}\frac{\overset{L}{\underset{j=1}{\sum }}{\left(\bar{y}\left(j\right)-y\left(j\right)\right)}^{2}}{\sigma^{2}}$$

where $\bar{y}\left(j\right)$ is the target value, *y*(*j*) is the predicted value, *L* is the total number of experimental data, and *σ* is the standard deviation of the target value. For the Santa Fe chaotic time series prediction task, when NMSE = 1, it represents that the RC system is completely unable to predict the next output of the chaotic sequence; when NMSE = 0, it represents that the RC system can accurately predict the next output of the chaotic sequence; Generally speaking, when NMSE ≤ 0.1, the predictive performance of RC systems can be considered good.

On the other hand, the channel equalization task (N task) is of significant importance in telecommunications. The goal of this task is usually to reconstruct the original signal *d*(*j*) propagated through a nonlinear channel from an observed signal *O*(*j*). The original signal *d*(*j*) is a random sequence of values taken in {−3; −1; 1; 3} [22]. The sequence first becomes *T*(*j*) through a linear channel, as shown below:

(2) $$\begin{array}{ll} T\left(j\right) & =0.08d\left(j+2\right)-0.12d\left(j+1\right)+d\left(j\right)+0.18d\left(j-1\right)\\ & \;-0\text{.}1d\left(j-2\right)+0.091d\left(j-3\right)-0.05d\left(j-4\right)\\ & \;+0.04d\left(j-5\right)+0.03d\left(j-6\right)+0.01d\left(j-7\right) \end{array}$$

Then, *T*(*j*) through the action of a nonlinear function, it becomes:

(3) $$O\left(j\right)=T\left(j\right)+0.026T{\left(j\right)}^{2}-0.11T{\left(j\right)}^{3}+v\left(j\right)$$

where *v*(*j*) is independent Gaussian white noise with a mean of 0, where $\left\langle v\left(t\right)v^{*}\left(t^{\prime }\right)\right\rangle =D_{e}\delta \left(t-t^{\prime }\right)$ . Usually through the selection of *D*_*e*_, making the signal-to-noise ratio (SNR) between 12 dB and 32 dB. Here, we set the SNR of the input signal of N task to 32 dB.

The symbol error rate (SER) is used as a performance evaluation indicator for this task, as follows [22]:

(4) $$\text{SER}=\frac{\text{Number}\;\;\text{of}\;\;\text{incorrect}\;\;\text{recognition}\;\;\text{values}}{\text{Total}\;\;\text{number}\;\;\text{of}\;\;\text{test}\;\;\text{values}}$$

When SER = 0, representing RC system reaches a perfect level in classification tasks and can successfully recover every original signal. Besides, we also adopt 3000 points for training and 1000 points for testing.

Here, we consider three injection cases: case 1-injecting two different Santa Fe chaotic time series prediction tasks and two different channel equalization tasks, denoted as S_1_N_2_S_3_N_4_; case 2-injecting four different Santa Fe chaotic time series prediction tasks (the four different Santa Fe chaotic time series prediction tasks are selected from different time periods from the Santa-Fe data set), denoted as S_1_S_2_S_3_S_4_; case 3-injecting four different channel equalization tasks, denoted as N_1_N_2_N_3_N_4_. Here, the case 2 S_1_S_2_S_3_S_4_, case 3 N_1_N_2_N_3_N_4_ stand for different samples data in the same task, respectively.

## 3 Experimental results

**Figure 2:**
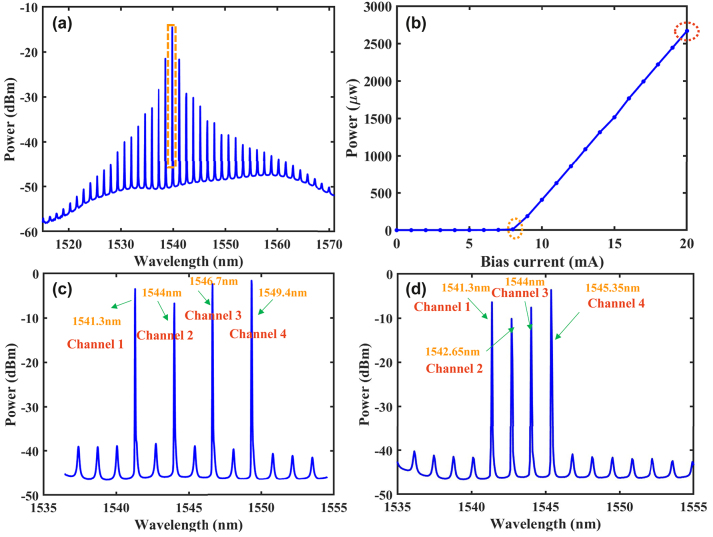
Basic characteristics of FP laser. (a) The optical spectrum of the free-running FP laser, when the temperature is fixed at 25 °C, and the bias current is 9.2 mA; (b) the PI curve of FP laser, and the temperature is stabilized at 25 °C; (c) the optical spectrum for the optically injected FP laser, when the wavelength interval is 2.7 nm; (d) the optical spectrum for the optically injected FP laser, when the wavelength interval is 1.35 nm.

The optical spectrum of the free-running FP laser is displayed in Figure 2(a). Here, the temperature is fixed at 25 °C, and the bias current is 9.2 mA. It can be seen that the FP laser exhibits multiple longitudinal modes with a center wavelength *λc* of 1539.91 nm. The spacing between the two longitudinal modes is approximately 1.35 nm. Besides, the power-current (PI) curve for the FP laser is presented in Figure 2(b). It can be observed that the threshold of the FP laser is approximately 8 mA. When the bias current is set as 20 mA, the power of FP laser is about 2.667 mW. In addition, the optical spectrums for the optically injected FP laser are shown in Figure 2(c) and (d). Obviously, four distinct peaks can be observed in the optical spectrum. For Figure 2(c), the wavelength values of the four distinct peaks are 1541.3 nm, 1544 nm, 1546.7 nm, and 1549.4 nm, representing four multiple tasks processing channels. And the wavelength interval between two adjacent distinct peak is 2.7 nm which is twice the interval between the two longitudinal modes. For Figure 2(d), the wavelength values of the four distinct peaks are 1541.3 nm, 1542.65 nm, 1544 nm, and 1545.35 nm, also standing for four multiple tasks processing channels. And the wavelength interval between two adjacent distinct peak is 1.35 nm which is equal to the interval between the two longitudinal modes.

**Figure 3:**
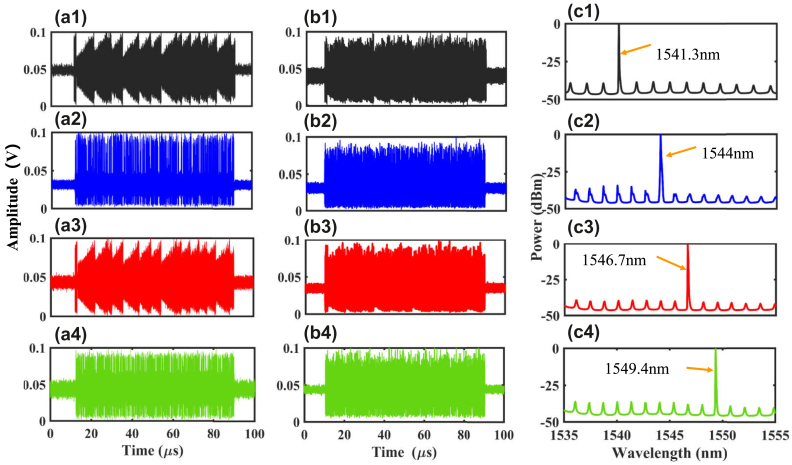
Four channel system input/output signals and the output optical spectrum after filtering the wavelength interval = 2.7 nm. (a1)–(a4) Input time series for case 1 S_1_N_2_S_3_N_4_; (b1)–(b4) output response for case 1 S_1_N_2_S_3_N_4_; (c1)–(c4) the optical spectrum of four channels after filtering, when the wavelength interval between adjacent channels is 2.7 nm and the bandwidth of the tunable optical filter is set to be 0.3 THz.

Here, we consider the injection case 1, with the injected signals being S_1_N_2_S_3_N_4_, and four injection wavelengths are 1541.3 nm, 1544 nm, 1546.7 nm, and 1549.4 nm. The optical spectrum for the optically injected FP laser is shown as Figure 2(c). Figure 3(a1)–(a4) represent the input time series for the four input signals, while Figure 3(b1)–(b4) show the output responses of the FP laser to the four input signals. It can be observed that the output responses match the inputs, but the output responses are more complex compared to the inputs. Note, the difference between input time series and output responses is partly due to the nonlinear effect of the FP laser, and partly due to the output response of the FP laser being amplified by an EDFA and photoelectric conversion of the PDs. Thus, EDFA and PDs both introduce some noise. We feed the response output of the FP laser into a multi-channel tunable optical filter, with each channel setting the bandwidth to be 0.3 THz and centre wavelengths setting to be 1541.3 nm, 1544 nm, 1546.7 nm, and 1549.4 nm, respectively. The filtering results for the four channels are shown in Figure 3(c1)–(c4). As shown in Figure 3(c1), a distinct peak at a wavelength of 1541.3 nm can be observed in the output spectrum of the first channel. Similarly, in Figure 3(c2)–(c4), noticeable peaks can be seen with center wavelengths of 1544 nm, 1546.7 nm, and 1549.4 nm, respectively. The results represent that the responses of the four channels can be effectively filtered out.

**Figure 4:**
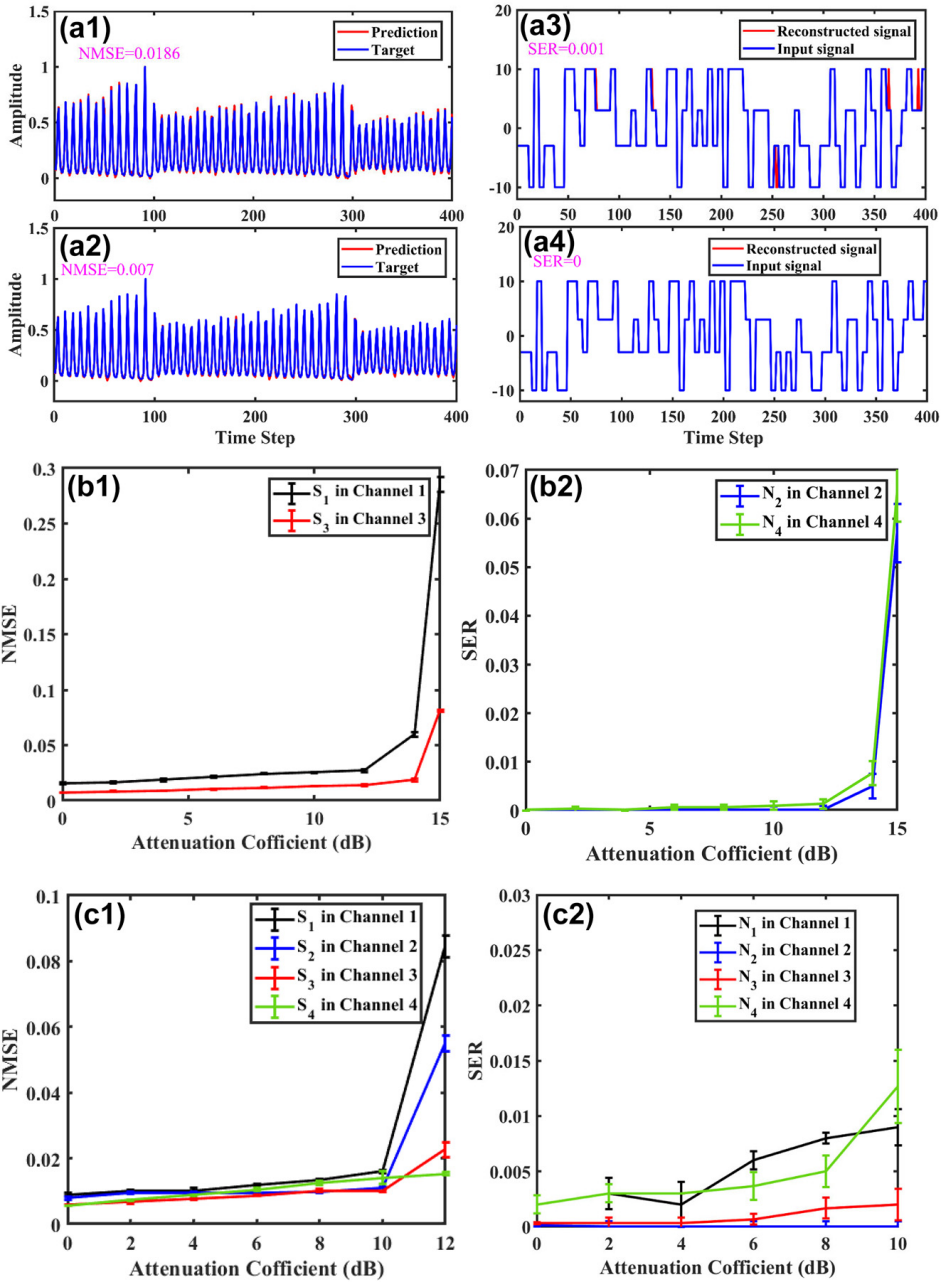
The effect of injection power on system performance with the wavelength interval of 2.7 nm. (a)The case 1 S_1_N_2_S_3_N_4_: time series of target and predicted values for Santa Fe time series prediction tasks (a1) for S_1_ task: NMSE = 0.0186; (a2) for S_3_ task: NMSE = 0.007; reconstruction targets and input signals for channel equalization tasks (a3) for N_1_ task: SER = 0.001; (a4) for N_2_ task: SER = 0; (b1) the NMSE values and (b2) the SER values obtained from the time delay RC system based on the FP laser for multiple tasks processing as a function of injection power for the case 1 S_1_N_2_S_3_N_4_; (c1) the NMSE values obtained from the time delay RC system based on the FP laser for multiple tasks processing as a function of injection power for the case 2 S_1_S_2_S_3_S_4_; (c2) the SER values obtained from the time delay RC system based on the FP laser for multiple tasks processing as a function of injection power for the case 3 N_1_N_2_N_3_N_4_.

When the injection power is almost 360 μw for four channels and the bias current is 9.2 mA of the FP laser, the multiple tasks processing performance of the time delay RC system based on the FP laser is analysed. On one hand, it can be seen from Figure 4(a1) and (a2), for S_1_ and S_3_ tasks, the red sequences represent the predicted values obtained after processing by the RC system, while the blue sequences represent the target values. It is obvious that the predicted values basically coincide with the target values. Besides, the NMSE value is 0.0186 for S_1_ task, and the NMSE value is 0.007 for S_3_ task. The performance obtained is better than that of some other papers [37], [39], [40], [41], which may be related to our parameter selection, the use of high-performance oscilloscopes and tunable optical filter to reduce noise, and the sacrifice of processing speed for performance improvement. On the other hand, as can be seen from Figure 4(a3) and (a4), for N_2_ and N_4_ tasks, the red sequences represent the reconstruction targets obtained after processing by the RC system, while the blue sequences represent the input signals. Similarly, it can be observed that the reconstructed signals and input signals basically coincide. Additionally, the SER value is 0.001 for N_2_ task, and the SER value is 0 for N_4_ task. Compared to the SER = 1.5 × 10^−2^ in Ref. [44], SER = 1 × 10^−4^ in Ref. [45], SER = 8 × 10^−4^ in Ref. [20], SER = 6 × 10^−4^ in Ref. [46], SER = 4 × 10^−4^ in Ref. [26], the performance we obtained is slightly better, which may also be due to our system itself, output layer data collection and processing, finding the optimal parameters, and the high SNR of the N task we used, and so on. Overall, the results indicate that the time delay RC system based on the FP laser has good multiple tasks processing performance.

Figure 4(b1) and (b2) further show the impact of the injection power on the multiple tasks processing performance of the time delay RC system based on the FP laser for the case 1 S_1_N_2_S_3_N_4_. Here, the initial injection power is fixed at 360 μw. Note that the results (including NMSE and SER values) shown in the following are the mean values over 5 runs, and the standard deviation around the mean value for the 5 runs is indicated by the vertical bars. AS can be seen from Figure 4(b1), for both S_1_ and S_3_ tasks which processed separately in channel 1 and channel 3, the NMSE values generally increase with the decreasing of the external injection power. For S_1_ task in channel 1, the lowest NMSE is 0.016. And for S_3_ task in channel 3, the lowest NMSE is 0.0068. This is because a large external injection power can achieve injection locking, which helps the time delay RC system based on the FP laser to maintain a steady state. For both N_2_ and N_4_ tasks which processed separately in channel 2 and channel 4, the SER values increase as the injection power decrease in Figure 4(b2). In addition, the lowest SER is 0 for both N_2_ and N_4_ tasks in channel 2 and channel 4. It means that the time delay RC experiment system can achieve high-quality multiple tasks processing performance within a wide range of the external injection power, and the performance can be comparable to or even better than those in Refs. [20], [26], [37], [39], [40], [41], [44], [45], [46].

Additionally, we also consider to process the same type of tasks in the four channels mentioned in Figure 2(c) of the FP laser, for instance, the case 2 S_1_S_2_S_3_S_4_ or the case 3 N_1_N_2_N_3_N_4_. Here, the initial injection power is fixed at 270 μw. On one hand, it can be seen from Figure 4(c1), for the case 2 S_1_S_2_S_3_S_4_, as the attenuation coefficient of injected power increases, the system multiple tasks processing performance gradually deteriorates. The lowest value of the case 2 S_1_S_2_S_3_S_4_ for channels 1, 2, 3, and 4 can reach 0.0093, 0.0085, 0.0057, and 0.0056, respectively (Due to line loss and the impact of noise, there are differences in the performance of the four channels). On the other hand, as can be seen from Figure 4(c2), for the case 3 N_1_N_2_N_3_N_4_, similar results can be observed. The SER values increase as the injection power decrease. The best performance with SER = 0.004, 0, 0, and 0.003 can be obtained for the case 3 N_1_N_2_N_3_N_4_, respectively. The experimental results demonstrate that in the four channels of the FP laser, it is possible to successfully parallelly accomplish both similar and dissimilar tasks, and the performance has demonstrated superiority.

**Figure 5:**
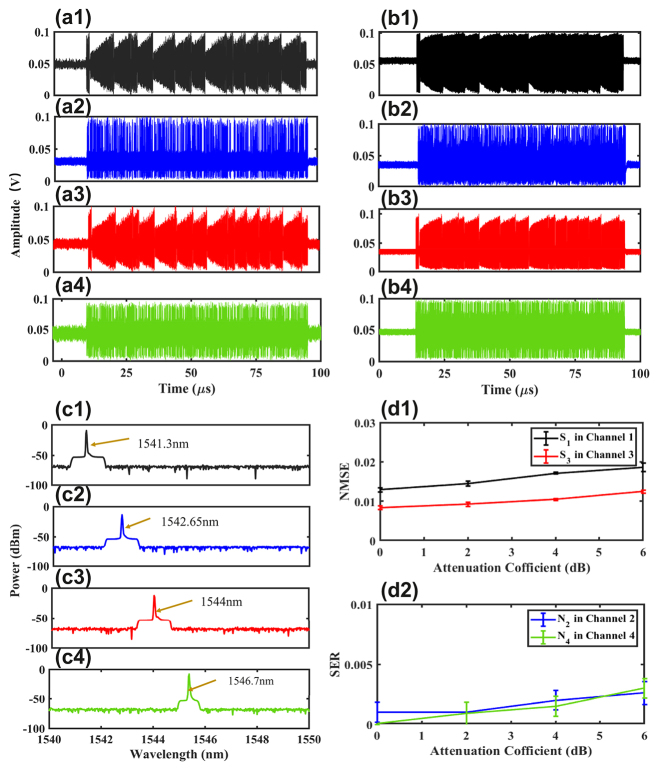
Four channel system input/output signals, the output optical spectrum after filtering the wavelength interval = 1.35 nm, and the effect of injection power on system performance when processing S_1_N_2_S_3_N_4_. (a1)–(a4) Input time series for the case 1 S_1_N_2_S_3_N_4_; (b1)–(b4) output response for the case 1 S_1_N_2_S_3_N_4_; (c1)–(c4) the optical spectrums of four channels after filtering, when the wavelength interval between adjacent channels is 1.35 nm and the bandwidth of the tunable optical filter is set to be 0.1 THz; (d1) the NMSE values and (d2) the SER values obtained from the time delay RC system based on the FP laser for multiple tasks processing as a function of injection power for the case 1 S_1_N_2_S_3_N_4_, when the filter bandwidth is set to 0.1 THz.

We further consider the ability of multiple tasks processing in the continuous four channels within the FP laser. When four tasks are injected into the FP laser, the optical spectrum is shown as Figure 2(d). The external injection wavelengths are set to be 1541.3 nm, 1542.65 nm, 1544 nm, and 1545.35 nm. The four wavelengths of the tunable optical filter are also configured to match the four external inputs, with each filter channel having a bandwidth of 0.1 THz. Here, we continue to use the case 1 S_1_N_2_S_3_N_4_ to validate the multiple tasks processing performance of the time delay RC system based on the FP laser. The injected signal for the case 1 S_1_N_2_S_3_N_4_ is shown in Figure 5(a1)–(a4) and (b1)–(b4) display the output after passing through the four channels of filter separately. It can be observed that the input and output share consistent profiles, but the output is more complex compared to the input. Figure 5(c1)–(c4) present the optical spectrum after filtering from the four channels of the FP laser.

For the case 1 S_1_N_2_S_3_N_4_, the NMSE values and the SER values obtained from the time delay RC system based on the FP laser for multiple tasks processing as a function of injection power are shown in Figure 5(d1) and (d2). Here, the filter bandwidth is set to 0.1 THz. It can be seen that as the attenuation coefficient of injected power increases, the system multiple tasks processing performance gradually deteriorates. The best performance of the S_1_ task and S_3_ task in channel 1 and channel 3 can reach NMSE = 0.013 and NMSE = 0.0083, respectively, while those of the N_2_ task and N_4_ task in channel 2 and channel 4 can achieve SER = 0.001 and SER = 0.0.

**Figure 6:**
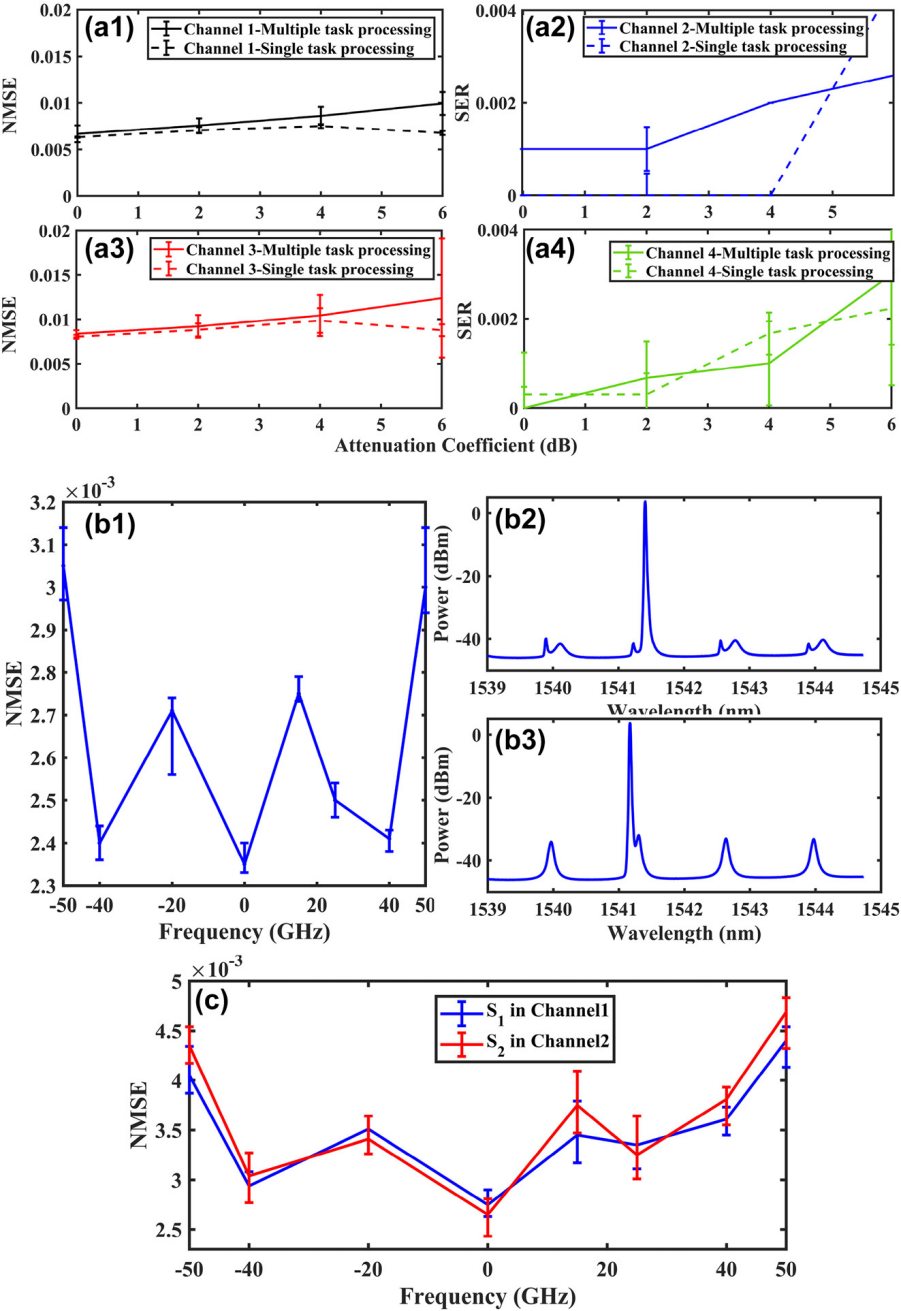
The NMSE values obtained from the time delay RC system based on the FP laser as a function of injection power for single and multiple tasks processing for (a1) channel 1, (a3) channel 3; the SER values obtained from the time delay RC system based on the FP laser as a function of injection power for single and multiple tasks processing for (a2) channel 2, (a4) channel 4; (b1) the NMSE values obtained from the time delay RC system based on the FP laser as a function of frequency detuning for single task processing; (b2) the optical spectrum for the optically injected FP laser when the frequency detuning Δ*f* is −10 GHz; (b3) the optical spectrum for the optically injected FP laser when the frequency detuning Δ*f* is 10 GHz; (c) the NMSE values obtained from the time delay RC system based on the FP laser as a function of frequency detuning for multiple tasks processing.

Without losing the generality, we conduct a comparative study of single task processing performance and multiple tasks processing performance in the time delay RC system based on the FP laser. Here, the injection power is kept almost same for each channel both for single task processing and multiple tasks processing. Moreover, for multiple tasks processing, the injection case 1 S_1_N_2_S_3_N_4_ is involved. While for single task processing, only S_1_ task is injected into one of the four channels, leaving the other channels unaffected. As shown in Figure 6(a1), it can be observed that for both single task processing and multiple tasks processing, good prediction performance of S_1_ task can be accomplished in channel 1. Furthermore, the NMSE value for single task processing is slightly lower than that for multiple tasks processing, indicating that prediction performance of single task processing is slightly superior to that of multiple tasks performance. As shown in Figure 6(a2), it can be observed that for the considered range of injection power, the SER value of N_2_ task for single task processing is generally lower than that for multiple tasks processing. Additionally, as can be seen from Figure 6(a3) channel 3 exhibits a similar trend to channel 1, with single task processing performance being superior to multiple tasks processing performance. For channel 4, single task processing performance is roughly equal to multiple task processing performance in Figure 6(a4). The results suggest that compared to single task processing, the performance degradation of multi tasks processing is not significant, with single task processing performance slightly better than multiple tasks processing performance, possibly due to the relatively small crosstalk between adjacent channels in the time delay RC system based on the FP laser during multiple tasks processing.

Note that we also utilize some proactive measures to reduce crosstalk between channels, such as: (a) in our experiment, we used a four-channel tunable light source to generate four independent carriers for carrying four independent mask input signals, thereby reducing the non-linear effects between carriers; (b) The frequency interval between adjacent channels of the FP laser we used in the experiment is about 168.75 GHz (the wavelength interval of the FP laser is about 1.35 nm). A larger wavelength interval can reduce the mutual interference between optical signals and the influence of cross gain modulation; (c) In the experiment, the modulation amplitude of our four shielded input signals is fixed, and each shielded input is injected with low optical power (the injection power of each channel remains almost unchanged), which can reduce the nonlinear effects caused by optical signals and thus reduce the degree of cross gain modulation; (d) In addition, in our system, we use erbium-doped fiber amplifiers (EDFAs) with uniform pump power distribution, which can reduce mutual modulation between signals; (e) Finally, we adopted a high-performance tunable optical filter (WaveShaper 16000A). The WaveShaper 16000A is a reconfigurable optical processor used as a programmable wavelength selective MxN filter. The introduction of optical filters can selectively block or attenuate signals within a specific wavelength range, thereby suppressing cross gain modulation.

Finally, the NMSE values as a function of frequency detuning for single task processing are further presented in Figure 6(b1). It is found that the prediction performance of the time delay RC system based on the FP laser is better when frequency detuning $\Delta f\in \left(-40\;\text{GHz},40\;\text{GHz}\right)$ , and the prediction performance deteriorates when the frequency detuning Δ*f* is less than −40 GHz or greater than 40 GHz. This indicates that within a large frequency detuning range, the time delay RC system based on the FP laser can achieve good prediction performance. Besides, the optical spectrums for the optically injected FP laser when the frequency detuning Δ*f* = −10 GHz and Δ*f* = 10 GHz are shown in Figure 6(b2) and (b3), respectively.

Besides, we also consider the impact of frequency detuning during the multiple tasks of the RC system. Here, we consider the case that the S_1_ task is injected to the channel 1 and the S_2_ task is injected to the channel 2. The NMSE values as a function of frequency detuning for multiple tasks processing are further presented in Figure 6(c). Note, the change in frequency detuning for both S_1_ and S_2_ tasks is consistent. It is found that when both tasks keep zero frequency detuning, the RC system achieves the best predictive performance. As the frequency detuning increases, the overall predictive performance shows a downward trend, which agrees with the results in Ref. [39].

## 4 Conclusions

In summary, a time delay reservoir computing system based on a FP laser for multiple tasks processing has been proposed, and it has been experimentally verified. Here, the various tasks have been attempted to perform in parallel in the multiple longitudinal modes of the FP lasers. Given the limitations of existing fiber optic platforms, we have considered three injection cases, including the case 1 S_1_N_2_S_3_N_4_, the case 2 S_1_S_2_S_3_S_4_ and the case 3 N_1_N_2_N_3_N_4_. Besides, two different adjacent information processing channel intervals, including 1.35 nm and 2.7 nm, are considered. It is found that the time delay RC system based on the FP laser could successfully handle different tasks across multiple longitudinal modes simultaneously for all the considered conditions. The experimental results demonstrated the potential of the time delay RC system based on FP lasers to achieve multiple tasks processing, providing various possibilities for improving the information processing ability of neural morphology RC systems, and promoting the development of RC systems.

In addition, in the experiment, we also found that the crosstalk effect between channels varies when processing different tasks. The crosstalk effect between channels may exhibit varying degrees of impact for different tasks. This may depend on various factors, such as the size of crosstalk between channels, the type of task processing in the channels, and channels noise. This is worth further in-depth research in next steps of work, and is crucial for further exploring the parallel processing performance and processing rate improvement of reserve pool computing systems based on multimode lasers.

## References

[1] Jaeger H. (2001). *The “Echo State” Approach to Analysing and Training Recurrent Neural Networks-With an Erratum Note*.

[2] Maass W., Natschläger T., Markram H. (2002). Real-time computing without stable states: a new framework for neural computation based on perturbations. *Neural Comput.*.

[3] Verstraeten D., Schrauwen B., D’Haene M., Stroobandt D. (2007). An experimental unification of reservoir computing methods. *Neural Netw.*.

[4] Freiberger M. A., Sackesyn S., Ma C., Katumba A., Dambre J. (2019). Improving time series recognition and prediction with networks and ensembles of passive photonic reservoirs. *IEEE J. Sel. Top. Quantum Electron.*.

[5] Lilak S. (2021). Spoken digit classification by in-materio reservoir computing with neuromorphic atomic switch networks. *Front. Nanotechnol.*.

[6] Liang X. (2022). Rotating neurons for all-analog implementation of cyclic reservoir computing. *Nat. Commun.*.

[7] Tian Y. (2022). Scalable and compact photonic neural chip with low learning-capability-loss. *Nanophotonics*.

[8] Clements W. R., Humphreys P. C., Metcalf B. J., Kolthammer W. S., Walmsley I. A. (2016). Optimal design for universal multiport interferometers. *Optica*.

[9] Vandoorne K. (2014). Experimental demonstration of reservoir computing on a silicon photonics chip. *Nat. Commun.*.

[10] Pathak S., Van Thourhout D., Bogaerts W. (2013). Design trade-offs for silicon-on-insulator-based AWGs for (de)multiplexer applications. *Opt. Lett.*.

[11] Donati G., Mirasso C. R., Mancinelli M., Pavesi L., Argyris A. (2022). Microring resonators with external optical feedback for time delay reservoir computing. *Opt. Express*.

[12] Gao C. (2023). Reservoir computing using arrayed waveguide grating. *2023 Opto-Electronics and Communications Conference (OECC)*.

[13] Borghi M., Biasi S., Pavesi L. (2021). Reservoir computing based on a silicon microring and time multiplexing for binary and analog operations. *Sci. Rep.*.

[14] Duport F., Schneider B., Smerieri A., Haelterman M., Massar S. (2012). All-optical reservoir computing. *Opt. Express*.

[15] Li X. (2023). Performance-enhanced time-delayed photonic reservoir computing system using a reflective semiconductor optical amplifier. *Opt. Express*.

[16] Van der Sande G., Brunner D., Soriano M. C. (2017). Advances in photonic reservoir computing. *Nanophotonics*.

[17] Bueno J., Brunner D., Fischer M. C. S., Fischer I. (2017). Conditions for reservoir computing performance using semiconductor lasers with delayed optical feedback. *Opt. Express*.

[18] Li S.-S. (2023). Photonic reservoir computing using a self-injection locked semiconductor laser under narrowband optical feedback. *Opt. Lett.*.

[19] Brunner D., Soriano M. C., Mirasso C. R., Fischer I. (2013). Parallel photonic information processing at gigabyte per second data rates using transient states. *Nat. Commun.*.

[20] Nguimdo R. M., Erneux T. (2019). Enhanced performances of a photonic reservoir computer based on a single delayed quantum cascade laser. *Opt. Lett.*.

[21] Hou Y. (2018). Prediction performance of reservoir computing system based on a semiconductor laser subject to double optical feedback and optical injection. *Opt. Express*.

[22] Sugano C., Kanno K., Uchida A. (2019). Reservoir computing using multiple lasers with feedback on a photonic integrated circuit. *IEEE J. Sel. Top. Quantum Electron.*.

[23] Guo X. X., Xiang S. Y., Zhang Y. H., Lin L., Wen A. J., Hao Y. (2020). High-speed neuromorphic reservoir computing based on a semiconductor nanolaser with optical feedback under electrical modulation. *IEEE J. Sel. Top. Quantum Electron.*.

[24] Huang Y., Zhou P., Yang Y., Chen T. (2021). Time-delayed reservoir computing based on a two-element phased laser array for image identification. *IEEE Photonics J.*.

[25] Huang Y., Zhou P., Yang Y. G., Li N. Q. (2022). Enhanced performance of reservoir computing using multiple self-injection and mutual injection vcsels. *IEEE J. Sel. Top. Quantum Electron.*.

[26] Nguimdo R. M., Verschaffelt G., Danckaert J., Van der Sande G. (2015). Simultaneous computation of two independent tasks using reservoir computing based on a single photonic nonlinear node with optical feedback. *IEEE Transact. Neural Networks Learn. Syst.*.

[27] Guo X. X., Xiang S. Y., Zhang Y. H., Lin L., Wen A. J., Hao Y. (2019). Polarization multiplexing reservoir computing based on a VCSEL with polarized optical feedback. *IEEE J. Sel. Top. Quantum Electron.*.

[28] Guo X. X., Xiang S. Y., Qu Y., Han Y. N., Wen A. J., Hao Y. Enhanced prediction performance of a neuromorphic reservoir computing system using a semiconductor nanolaser with double phase conjugate feedbacks. *J. Lightwave Technol.*.

[29] Sattar Z. A., Shore K. A. (2015). External optical feedback effects in semiconductor nanolasers. *IEEE J. Sel. Top. Quant. Electron.*.

[30] Jiang N. (2020). Simultaneous bandwidth-enhanced and time delay signature-suppressed chaos generation in semiconductor laser subject to feedback from parallel coupling ring resonators. *Opt. Express*.

[31] Li N., Susanto H., Cemlyn B., Henning I., Adams M. (2017). Stability and bifurcation analysis of spin-polarized vertical-cavity surface-emitting lasers. *Phys. Rev. A.*.

[32] Deng T., Robertson J., Hurtado A. (2017). Controlled propagation of spiking dynamics in vertical-cavity surface-emitting lasers: towards neuromorphic photonic networks. *IEEE J. Sel. Top. Quantum Electron.*.

[33] Zhao A., Jiang N., Peng J., Liu S., Zhang Y., Qiu K. (2022). Parallel generation of low-correlation wideband complex chaotic signals using CW laser and external-cavity laser with self-phase-modulated injection. *Opto-Electron. Adv.*.

[34] Tsang H. K., Chan L. Y., Yam S. P., Shu C. (1998). Experimental characterization of dual-wavelength injection-locking of a Fabry–Perot laser diode. *Opt. Commun.*.

[35] Tromborg B., Olesen H., Pan X., Saito S. (1987). Transmission line description of optical feedback and injection locking for Fabry–Perot and DFB lasers. *IEEE J. Quantum Electron.*.

[36] Bogris A., Mesaritakis C., Deligiannidis S., Li P. (2020). Fabry–Perot lasers as enablers for parallel reservoir computing. *IEEE J. Sel. Top. Quantum Electron.*.

[37] Harkhoe K., Sande G. V. d. (2019). Delay-based reservoir computing using multimode semiconductor lasers: exploiting the rich carrier dynamics. *IEEE J. Sel. Top. Quantum Electron.*.

[38] Skontranis M., Sarantoglou G., Sozos K., Kamalakis T., Mesaritakis C., Bogris A. (2023). Multimode Fabry–Perot laser as a reservoir computing and extreme learning machine photonic accelerator. *Neuromorph. Comput. Eng.*.

[39] Harkhoe K., Sande G. V. D., Katumba A., Bienstman P., Van der Sande G. (2020). Demonstrating delay-based reservoir computing using a compact photonic integrated chip. *Opt. Express*.

[40] Hou Y. S. (2018). Prediction performance of reservoir computing system based on a semiconductor laser subject to double optical feedback and optical injection. *Opt. Express*.

[41] Kuriki Y., Nakayama J., Takano K., Uchida A. (2018). Impact of input mask signals on delay-based photonic reservoir computing with semiconductor lasers. *Opt. Express*.

[42] Weigend A. S. (2018). *Time Series Prediction: Forecasting the Future and Understanding the Past*.

[43] Nguimdo R. M., Verschaffelt G., Danckaert J., Van der Sande G. (2014). Fast photonic information processing using semiconductor lasers with delayed optical feedback: role of phase dynamics. *Opt. Express*.

[44] Vatin J., Rontani D., Sciamanna M. (2019). Experimental reservoir computing using VCSEL polarization dynamics. *Opt. Express*.

[45] Vatin J., Rontani D., Sciamanna M. (2018). Enhanced performance of a reservoir computer using polarization dynamics in VCSELs. *Opt. Lett.*.

[46] Nguimdo R. M., Verschaffelt G., Danckaert J., Van der Sande G. (2016). Reducing the phase sensitivity of laser-based optical reservoir computing systems. *Opt. Express*.

