# Corrigendum to: Experimental demonstration of a photonic reservoir computing system based on Fabry Perot laser for multiple tasks processing

**DOI:** 10.1515/nanoph-2024-0713

**Published:** 2025-02-14

**Authors:** Xingxing Guo, Hanxu Zhou, Shuiying Xiang, Qian Yu, Yahui Zhang, Yanan Han, Tao Wang, Yue Hao

**Affiliations:** State Key Laboratory of Integrated Service Networks, Xidian University, Xi’an 710071, China; State Key Discipline Laboratory of Wide Bandgap Semiconductor Technology, School of Microelectronics, Xidian University, Xi’an 710071, China

This corrigendum corrects errors in the Funding statement of the original paper, *Nanophotonics*, vol. 13, no. 9, pp. 1569–1580, 2024, https://doi.org/10.1515/nanoph-2023-0708.

Errors have been found in the funding information indicated in our paper [1].

The Funding statement is corrected as follows:
